# A multi-centric evaluation of self-learning GAN based pseudo-CT generation software for low field pelvic magnetic resonance imaging

**DOI:** 10.3389/fonc.2023.1245054

**Published:** 2023-11-10

**Authors:** Jessica Prunaretty, Gorkem Güngör, Thierry Gevaert, David Azria, Simon Valdenaire, Panagiotis Balermpas, Luca Boldrini, Michael David Chuong, Mark De Ridder, Leo Hardy, Sanmady Kandiban, Philippe Maingon, Kathryn Elizabeth Mittauer, Enis Ozyar, Thais Roque, Lorenzo Colombo, Nikos Paragios, Ryan Pennell, Lorenzo Placidi, Kumar Shreshtha, M. P. Speiser, Stephanie Tanadini-Lang, Vincenzo Valentini, Pascal Fenoglietto

**Affiliations:** ^1^ Institut du Cancer de Montpellier, Department of Radiation Oncology, Montpellier, France; ^2^ Department of Radiation Oncology, Maslak Hospital, Acibadem Mehmet Ali Aydınlar (MAA) University, Istanbul, Türkiye; ^3^ Radiotherapy Department, Universitair Ziekenhuis (UZ) Brussel, Vrije Universiteit Brussel, Brussels, Belgium; ^4^ Department of Radiation Oncology, University Hospital Zurich, Zurich, Switzerland; ^5^ Radiation Oncology, Fondazione Policlinico Universitario A. Gemelli IRCCS, Rome, Italy; ^6^ Department of Radiation Oncology, Miami Cancer Institute, Miami, FL, United States; ^7^ TheraPanacea, Paris, France; ^8^ Assistance publique – Hôpitaux de Paris (AP-HP) Sorbonne Universite, Charles-Foix Pitié-Salpêtrière, Paris, France; ^9^ Department of Radiation Oncology, Miami Cancer Institute, Baptist Health South Florida, Miami, FL, United States; ^10^ Radiation Oncology, NewYork-Presbyterian/Weill Cornell Hospital, New York, NY, United States; ^11^ Radiation Oncology Weill Cornell Medicine, New York, NY, United States

**Keywords:** pseudo-CT, artificial intelligence, MRI, pelvis, cycle GAN

## Abstract

**Purpose/objectives:**

An artificial intelligence-based pseudo-CT from low-field MR images is proposed and clinically evaluated to unlock the full potential of MRI-guided adaptive radiotherapy for pelvic cancer care.

**Materials and method:**

In collaboration with TheraPanacea (TheraPanacea, Paris, France) a pseudo-CT AI-model was generated using end-to-end ensembled self-supervised GANs endowed with cycle consistency using data from 350 pairs of weakly aligned data of pelvis planning CTs and TrueFisp-(0.35T)MRIs. The image accuracy of the generated pCT were evaluated using a retrospective cohort involving 20 test cases coming from eight different institutions (US: 2, EU: 5, AS: 1) and different CT vendors. Reconstruction performance was assessed using the organs at risk used for treatment. Concerning the dosimetric evaluation, twenty-nine prostate cancer patients treated on the low field MR-Linac (ViewRay) at Montpellier Cancer Institute were selected. Planning CTs were non-rigidly registered to the MRIs for each patient. Treatment plans were optimized on the planning CT with a clinical TPS fulfilling all clinical criteria and recalculated on the warped CT (wCT) and the pCT. Three different algorithms were used: AAA, AcurosXB and MonteCarlo. Dose distributions were compared using the global gamma passing rates and dose metrics.

**Results:**

The observed average scaled (between maximum and minimum HU values of the CT) difference between the pCT and the planning CT was 33.20 with significant discrepancies across organs. Femoral heads were the most reliably reconstructed (4.51 and 4.77) while anal canal and rectum were the less precise ones (63.08 and 53.13). Mean gamma passing rates for 1%1mm, 2%/2mm, and 3%/3mm tolerance criteria and 10% threshold were greater than 96%, 99% and 99%, respectively, regardless the algorithm used. Dose metrics analysis showed a good agreement between the pCT and the wCT. The mean relative difference were within 1% for the target volumes (CTV and PTV) and 2% for the OARs.

**Conclusion:**

This study demonstrated the feasibility of generating clinically acceptable an artificial intelligence-based pseudo CT for low field MR in pelvis with consistent image accuracy and dosimetric results.

## 1 Introduction

Magnetic resonance-guided radiotherapy (MRgRT) allows plan adaptation on the magnetic resonance imaging (MRI) of the day, offering new perspectives in pelvic cancer treatment. Besides structure tracking and automated beam gating, the MR-linac combination benefits a higher soft tissue contrast and allows on-table plan adaptation (1). Several studies have shown promising early results and a safe dose escalation using isotoxic approaches with stereotactic MR-guided adaptive radiation therapy (SMART) appears to improve disease outcomes across a range of tumor sites (2, 3).

However, the MRgRT suffers from a lack of correlation between MR intensities and electron densities (ED), requiring a planning CT acquisition for dose calculation (4). In the daily MRgRT process, a deformable image registration is applied to the planning CT from the day’s MRI to propagate the ED map. A user-defined density override is then performed to correct for daily air cavity variations. This approach is time consuming, especially in the pelvic region where the organ filling changes are recurrent, and penalizes the adaptive radiotherapy workflow. Furthermore, the manual corrections are subject to the operator interpretation, and introduce additional dosimetric uncertainties, especially in the presence of the magnetic field (5). The key was to substitute the planning CT with a pseudo-CT from the MR. Historically, the main approaches have been the bulk density assignment and the atlas-based method (6, 7). some solutions have been already commercialized (8, 9). With the development of artificial intelligence, studies have focused on deep learning approaches using multiple architectures (10). These approaches were initially developed from diagnostic (high field) MRI data (11–14). However, the low-field MR linac, the MRIDian (ViewRay, Inc., Oakwood Village, Ohio, USA), uses a True Fast Imaging with Steady State-Free Precession (TrueFISP) sequence for data acquisition (15). This sequence has a limited field of view and a lower signal-to-noise ratio, posing new challenges. To date, few results have been published on the generation of pseudo-CT from 0.35T MRI for the pelvic region (16–18). This study proposes and clinically evaluates an artificial intelligence-based pseudo-CT to overcome these challenges and unlock the full potential of MRgRT for pelvic cancer care.

## 2 Materials and methods

### 2.1 Deep learning workflow

In collaboration with TheraPanacea (TheraPanacea, Paris, France), a pseudo-CT AI model was generated using end-to-end ensembled self-supervised GANs endowed with cycle consistency using data from 350 pairs of weakly aligned data from pelvic planning CTs and TrueFisp (0.35T) MRIs. The first GANs were introduced by Goodfellow et al. to train generative models in an adversarial manner (19). These neural network training methods led to the introduction of conditional GANs (20) for image translation of paired images, where one input is translated into a different but perfectly paired one. Due to the limited availability of paired images in practice, Zhu et al. (21) introduced a CycleGAN that simultaneously learns two generators in a cyclic manner. This CycleGAN architecture opens up the possibility of translating one image into another even if the images are weakly paired, i.e. there are no voxel-to-voxel correspondences. We therefore exploit this potential to build a dataset of MRI, CT pairs.

**Figure 1:**
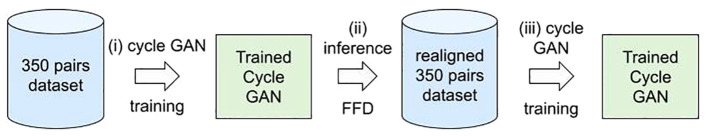
Flowchart of the training procedure.

The training procedure is shown in **Figure 1**. A two-step process was used, meaning that the cycle GANs that generate the synthetic CTs are trained twice. The first training is performed between the 350 pairs of weakly aligned data from the pelvic planning CTs and the TrueFisp (0.35T) MRIs. After this first step, the MR images used for training are converted to CT scans and aligned with the input CT scans based on deformable registration. The result of this registration is a new dataset of MR-CT pairs, where the pairs are much better aligned. Finally, a new cycle GAN is trained on this new dataset. This is the second training phase of our training procedure.

The training procedure is done first by gathering a curated dataset and then training the cycle GAN, usually for about two weeks of time, to ensure that during the training procedure, the GAN reaches an equilibrium, where the discriminator and the generator are on par.

The developed AI model is then used for the generation of pseudo-CTs from TrueFisp images of the MR-Linac on 4 Nvidia gtx 2080ti GPUS in parallel. The training time was 131h with a maximum GPU usage of 18721MB.

### 2.2 Image accuracy

A retrospective cohort of 20 test cases from eight different institutions (US: 2, EU: 5, AS: 1) and different CT manufacturers was used to evaluate the image accuracy of the generated pCT. The planning CTs were non-rigidly registered to the MRIs for each patient. These were termed warped CTs (wCT). Pseudo-CT images were compared with wCT images to assess reconstruction performance. A Hounsfield unit comparison was performed with the organs at risk used for treatment.

### 2.3 Dosimetric evaluation

Twenty-nine prostate cancer patients treated on the low-field MR linac (ViewRay) at the Montpellier Cancer Institute were selected. All patients underwent computed tomography (CT)-based simulation (Optima CT580 RT, General Electric Healthcare, Waukesha, WI). CT images were acquired with a slice thickness of 2.5 mm. The CT acquisition was followed by the MR simulation. The time interval between the two simulations was reduced as much as possible to avoid anatomical changes. The MR acquisition consisted of a true fast imaging with steady-state precession (TrueFISP) sequence performed with the same patient positioning setup. Acquisition parameters were 173 s, a resolution of 0.15 cm and a FOV of 50*45*43 cm.

Planning CTs were non-rigidly registered to the MRIs for each patient. Treatment plans were optimized on the planning CT with a clinical TPS fulfilling all clinical criteria and recalculated on the wCT and the pCT. Three different algorithms were used: AAA, AcurosXB and MonteCarlo. AAA and AXB dose calculations were performed with Eclipse TPS (version 15.6, Varian, Medical Systems, Palo Alto, CA, USA) and a volumetric modulated arc therapy (VMAT) geometry using a dose calculation grid size of 0.25cm. The MonteCarlo algorithm was used with Viewray TPS (version 5.4.1.34) considering the magnetic field presence, step-and-shoot intensity modulated radiation therapy (IMRT) beams and a dose calculation grid size of 0.3cm.

The dose distributions of the pseudo-CT and the distorted CT were compared using global gamma passing rates and dose metrics. Gamma analysis was performed using the 3%/3mm, 2%/2mm, 1%/1mm criterion and thresholds of 40% and 10%. For DVH parameters, reference structures were rigidly propagated from MRI to wCT and pCT. Dose metrics for the target volumes (CTV and PTV) were defined according to ICRU 83 recommendations (22). For OAR, D1%, D25% and D50% were evaluated for rectum and bladder.

## 3 Results

**Figure 2:**
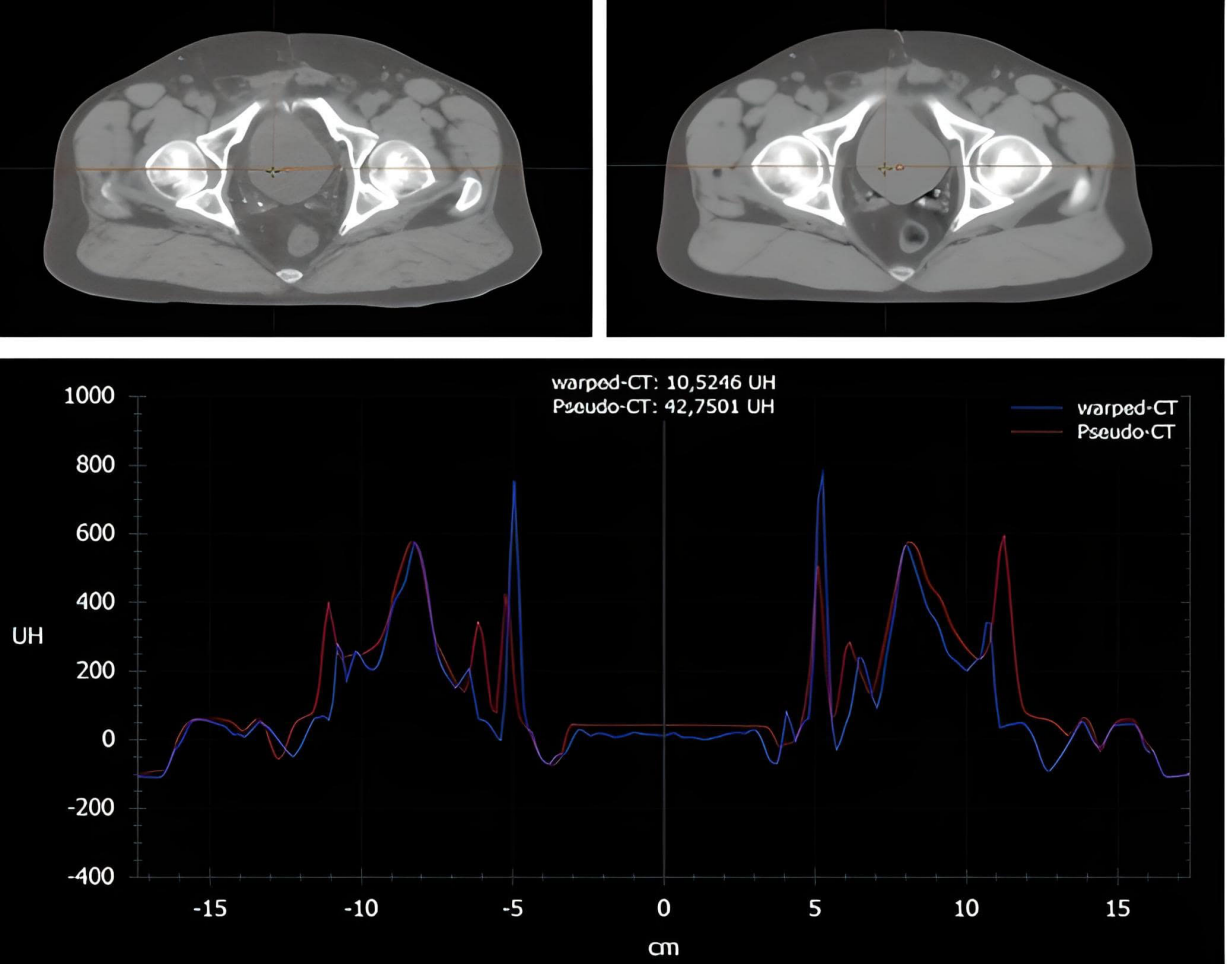
Example of Hounsfield unit comparison between the warped CT (left) and the pseudo-CT (right).

**Figure 2** shows an example of a pseudo-CT generated by the AI-based tool and compares the Hounsfield units with the corresponding warped CT. The time for running the sCT generation was 25s ± 4s. The HU comparison for each organ between the pseudo-CT and the warped CT is detailed in the **Table 1**. The observed average scaled (between the maximum and minimum HU values of the CT) difference between the pCT and the warped CT was 33.20 with significant discrepancies between organs. Femoral heads were reconstructed most reliably (4.51 & 4.77), while gastrointestinal organs were less accurate: 63.08, 53.13 and 51.48 for the anal canal, rectum and sigmoid, respectively.

**Table 1:** Comparison of mean HU between pseudo-CT and warped CT for each organ.

Organ/Reconstruction Error	Mean (de scaled diff min (%))	Means (warped CT)	Means (pseudo CT)
Anal canal	63.08	39.56	58.44
Bladder	20.62	16.94	32.06
Left femoral head	4.51	263.35	278.65
Penile bulb	47.62	38.67	50.58
Prostate	36.8	37.57	56.14
Rectum	53.13	-0.33	29.44
Right femoral head	4.77	256.1	280.1
Seminal vesicle	31.47	27.53	47.53
Sigmoid	51.48	-14.28	-1.53
***Total***	***33.20***	***85.30***	***104.08***

The mean global gamma analysis with three tolerance criteria (3%3mm, 2%2mm, 1%1mm) and two dose thresholds (10% and 40%) showed a good agreement between the dose distribution calculated on the pseudo-CT and the warped CT (**Table 2**). The minimum mean values were obtained with the AXB algorithm and 40% threshold: 99.88 ± 0.20%, 98.56 ± 1.59% and, 89.65 ± 8.17% for 3%3mm, 2%2mm, and 1%1mm criterion, respectively.

**Table 2:** Comparison of the gamma passing rates for AAA, AXB and EMC algorithms using 3%3mm, 2%2mm and 1%1mm criterion with 10% and 40% dose thresholds.

	Threshold 10%			*Threshold 40%*		
	3%3mm	2%2mm	1%1mm	3%3mm	2%2mm	1%1mm
**AAA**	99,88 ± 0,11	99,57 ± 0,32	99,04 ± 0,64	99,99 ± 0,03	99,86 ± 0,39	98,99 ± 2,78
**AXB**	99,86 ± 0,13	99,29 ± 0,48	96,57 ± 2,35	99,88 ± 0,20	98,56 ± 1,59	89,65 ± 8,17
**eMC**	99,99 ± 0,02	99,93 ± 0,13	96,87 ± 4,27	99,99 ± 0,02	99,69 ± 0,50	90,64 ± 11,01

**Figure 3:**
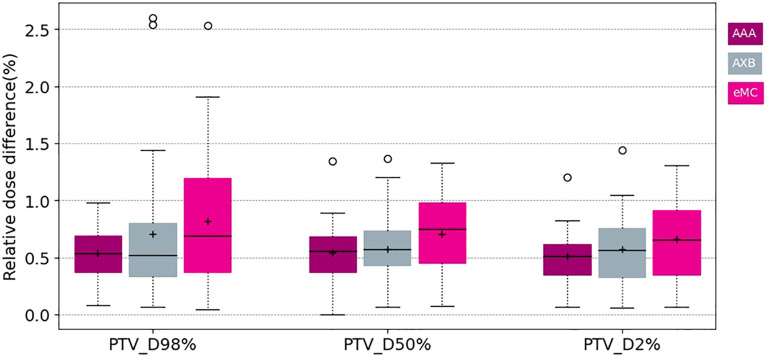
Boxplots of the relative dose difference of the D98 D50 and D2 for the PTV using different calculation algorithms AAA (purple) AXB (grey) and eMC (pink).

**Figure 4:**
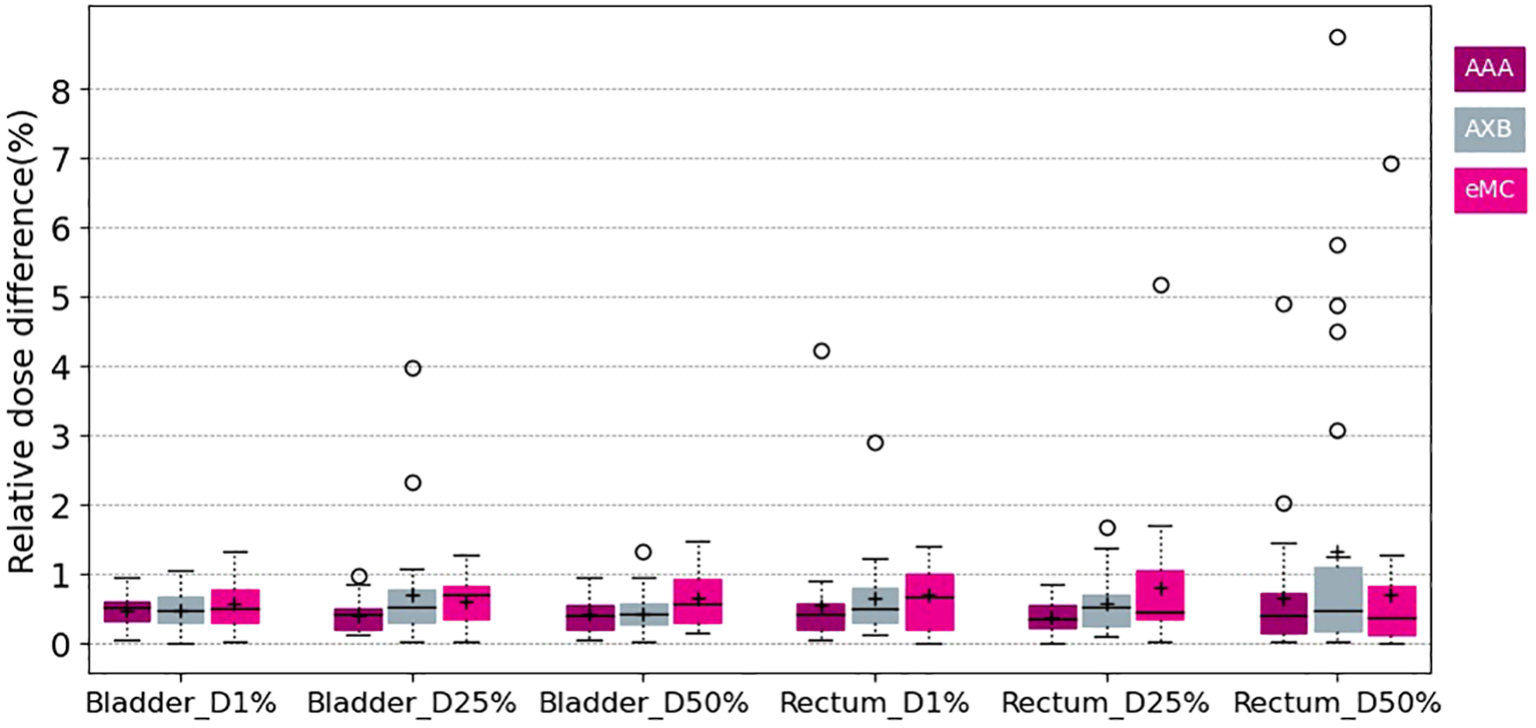
Boxplots of the relative dose difference of the D1% D25% and D50% for the bladder and the rectum using different calculation algorithms AAA (purple) AXB.

**Figure 5:**
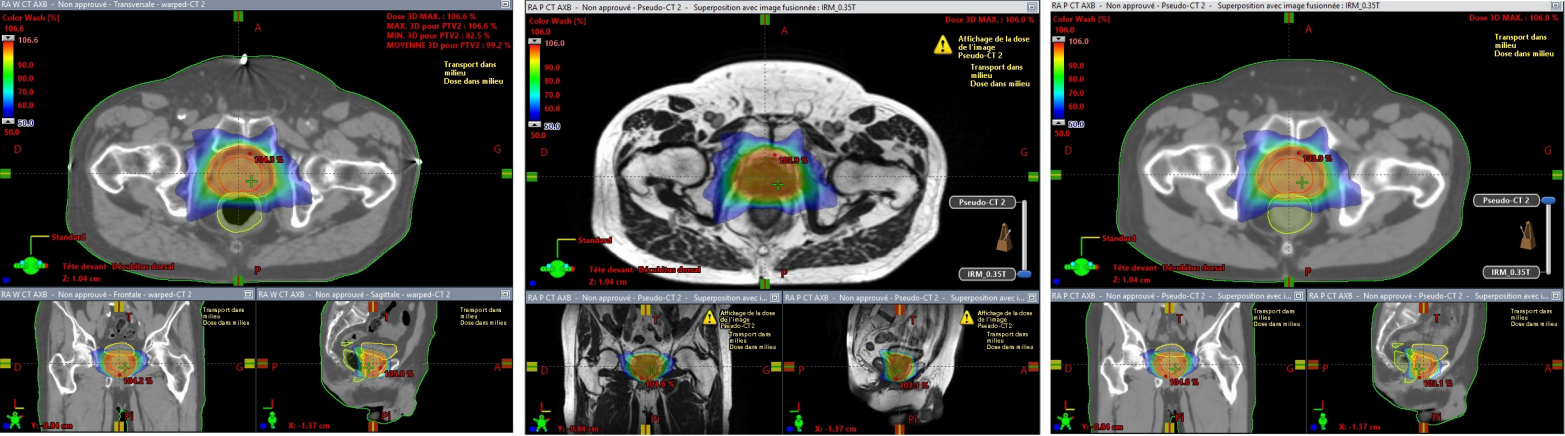
Patient data with the largest relative dose difference for D98% for the PTV using the AXB algorithm. Dose distribution (98% isodose) on wCT (left) MR (center) and pCT (right).

**Figures 3** and **4** show the relative dose difference for the PTV and the organs at risk between plans calculated using the pseudo-CT and the warped CT using the AAA, AXB and eMC algorithms. The median relative dose differences for the PTV were lower than 0.5% for each dose metric (D98%, D95%, D50%, and D2%) and algorithm. The maximum reported value was 2.60% for D98% using the AXB algorithm, equivalent to 2.03Gy. **Figure 5** shows the patient data with this largest relative dose difference.

Concerning the organs at risk, the median relative dose difference were less than 0.5%, regardless the dose metrics and algorithms assessed. However, outliers were more frequent, especially for the rectum. The maximal difference was 8.76% for D50% using the AXB algorithm, corresponding to 2.67Gy. **Figure 5** shows the patient data with this largest relative dose difference.

## 4 Discussion

A 3D cycle GAN model for pseudo-CT generation from low-field pelvic MRI was presented in this study. The strengths of this work were the multi-centric factor, which integrates several CT vendors with different image characteristics, and the large number of patients used in the training model, which ensures reliable pseudo-CT generation. Furthermore, the cycle GAN architecture does not require paired images. This is essential for pelvic localization, where anatomical variations are common.

Regarding the qualitative evaluation, the results obtained in this study are satisfying, showing a good agreement between the pseudo-CT and the warped CT. However, the presence of fiducial markers, as shown in **Figure 5**, severely penalizes reconstruction due to implicit error propagation associated with the “convolutional” nature of deep learning. The presence of air bubbles visible on the CT (sigmoid and rectum) is also a problem, as these elements are barely perceptible on the MR and are therefore difficult to predict by the generator. To limit these deviations, Cusumano et al. excluded some patients from their neural network training due to artefacts (artificial implants) or differences in air pocket locations between CT and MR images (17), while Maspero et al. trained their model by enforcing air consistency (13).

This work demonstrated that AI-driven pseudo-CT generation from low-field MRI was clinically accurate for the pelvic region. The dosimetric evaluation showed median dose differences within 0.5% and gamma pass rates greater than 99% (for 3%3mm and 2%2mm criteria) regardless of the algorithm used, thus meeting the clinical acceptance criteria (23). The previous studies for pelvic cancer using low field MRI and different deep learning based methods (17, 18) (24) published comparable results. Cusumano et al. (17) and Hsu et al. (18) developed conditional Generative Adversarial Networks and showed dose differences of less than 1% and gamma pass rates in a similar range. Nousiainen et al. (24) evaluated another pCT generation algorithm using a convolutional neural network based on HighRes3DNet for the abdominal region (including pelvic cancer). They reported an equivalent relative dose difference. With the exception of the outliers, the largest differences were obtained using the Monte Carlo algorithm with the presence of the magnetic field, which is known to have a significant dosimetric impact at the air-tissue interfaces due to inaccurate local electron density mapping (25). In addition to dosimetric accuracy, the quality of pseudo-CT for pre-treatment verification of patient position should be assessed for full clinical implementation of an MR-only pathway (26).

The main limitation of this study was the selection of a “ground truth” image to assess the quality of the pCTs generated from MRI. We chose a non-rigid registration of the planning CT which was defined as the reference CT. However, the delay between the MRI acquisitions and the planning CT leads to anatomical variations (organ filling, air cavity variations) and deviations between the two images. Therefore, as shown in **Figure 5**, outliers may occur due to a “wrong” reference CT compared to the MR data and not due to an inappropriate pCT. Then, in the MR-only objective, the positioning performance of sCT should be evaluated to facilitate the clinical integration of sCT for treatment planning and verification of patient positioning as in other studies (8) (27).

Finally, with the emergence of a variety of sCT generators using different deep learning-based methods, the implementation of a quality assurance process is essential to ensure the safe and reliable integration of deep learning into clinical workflow, ultimately improving the overall efficiency of MRI-guided radiotherapy by generating synthetic CT volumes from MRI data.

## 5 Conclusion

This retrospective multi-center study has demonstrated the potential of a fully low-field MR-based treatment planning workflow. This artificial intelligence-based tool can be considered clinically acceptable, while reducing imaging dose and registration issues, as it can be used in few seconds to generate a pseudo CT image, bypassing the need for a planning CT. In future work, the accuracy of the use of this pCT tool for MRgRT treatment of other anatomical regions will be investigated.

## Data availability statement

The raw data supporting the conclusions of this article will be made available by the authors, without undue reservation.

## Author contributions

JP wrote the manuscript. GG, TG, SV, PB, LB, MC, MR, PM, KM, EO, RP, LP, MS, ST-L, SV, and VV contributed to conception of the study. LH, SK, TR, LC, NP, and KS contributed to the conception and design of the study, performed the analysis, and wrote sections of the manuscript. PF contributed to the conception and design of the study and revised the manuscript. All authors contributed to the article and approved the submitted version.
